# Greater Attention to Gingival Cancer and Floor of Mouth Cancer: Based on a Retrospective Analysis of Oral Cancer Across Different Subsites

**DOI:** 10.1002/hed.70145

**Published:** 2025-12-22

**Authors:** Hao Li, Yi Ding, Yansheng Wu, Yan Fang, Xinhua Li, Weifeng Shi, Shaoshi Chen, Ailin Yang, Jin Zhang, Ruoyu Shi, Chao Jing, Xudong Wang, Yuansheng Duan

**Affiliations:** ^1^ Department of Head and Neck Oncology Tianjin Medical University Cancer Institute & Hospital Tianjin China; ^2^ Key Laboratory of Basic and Translational Medicine on Head & Neck Cancer Tianjin China; ^3^ National Clinical Research Center for Cancer, Tianjin's Clinical Research Center for Cancer Tianjin China

**Keywords:** floor‐of‐mouth, gingival, oral squamous cell carcinoma, prognosis, risk factor

## Abstract

**Background:**

The gingiva and floor of the mouth are distinct subsites, and oral squamous cell carcinoma (OSCC) originating in these locations warrants further indepth understanding.

**Methods:**

This study enrolled patients with OSCC from 2000 to 2020, and analyzed the clinicopathological characteristics. Kaplan–Meier analysis compared overall survival (OS) and recurrence‐free survival (RFS). Univariate analysis and multivariate Cox proportional hazards model examined the risk factors on survival outcomes.

**Results:**

A total of 885 OSCC patients were included, with the most common tumor subsites: oral tongue (41.9%), gingiva (31.8%), and floor of mouth (16.0%). Gingival and floor of mouth showed a higher proportion of advanced stage (65.8%, 66.9%) and significantly poorer OS (3‐year: 63.0%, 57.0%; 5‐year: 49.8%, 37.3%) and RFS (3‐year: 59.1%, 61.3%; 5‐year: 55.5%, 57.0%). Neoadjuvant chemotherapy was associated with significantly reduced mortality in gingival cancer (HR = 0.598), while tobacco exposure (HR = 1.952) was associated with a significant increase in mortality in floor of mouth cancer.

**Conclusion:**

Gingival cancer and floor of mouth cancer have a worse prognosis, and thus require greater attention throughout the entire management process.

## 1 Introduction

Oral cancer ranks as the 16th most prevalent cancer worldwide [1], and oral squamous cell carcinoma (OSCC) is the most common pathological type [2]. In China, the incidence of oral cancer ranks 19th among malignant tumors [3], and the incidence is increasing year by year. From 1990 to 2021, the number of male oral cancer patients in China increased by 341.20%, and that of female patients increased by 180.33%. It is estimated that by 2036, the number of male oral cancer patients will continue to increase by 52.25%, and that of female patients will increase by 85.27% [4]. As the burden of oral cancer on China gradually increases, there is a growing focus on its precise management of OSCC. Oral cancer can be classified into six subsite origins based on anatomical locations: buccal mucosa, floor of mouth, oral tongue, gingival, retromolar trigone, and hard palate [5]. However, the gingiva and floor of mouth differ from other oral subsites in terms of anatomy, histological structure, and microenvironment, and thus these two subsites exhibit distinct disease characteristics. For example, the mandibular gingiva is a mere 2 mm or so in thickness, rendering it highly susceptible to early bone invasion and progression to advanced disease stages [6]. The floor of mouth comprises loose connective tissue with interconnected anatomical spaces, along with a dense network of lymph nodes and nerves, features that facilitate the spread of tumors along these spaces [7, 8]. Thus, enhancing focus on these highly invasive subsites, analyzing their disease characteristics and prognostic factors, can provide a theoretical basis for the individualized management of patients with OSCC.

This study compared the clinicopathological features and prognosis of OSCC at our center, and analyzed the risk factors affecting the long‐term survival of patients with gingival cancer and floor of mouth cancer, thereby providing management recommendations for patients with OSCC in these subsites.

## 2 Methods

### 2.1 Design of the Study and Populations

This retrospective study enrolled patients initially diagnosed with primary OSCC at our institution who underwent surgical intervention between 2000 and 2020. Exclusion criteria included, unclear primary tumor site, involvement of multiple primary tumor sites, absence of surgical intervention, unclear treatment strategy, lack of intraoperative pathological data, or having other malignant tumors. The analysis considered patient demographics (age, sex, tobacco and alcohol consumption), tumor characteristics (primary subsite, TNM stage, depth of invasion, lymph node metastasis and grade of differentiation), and treatment details (neck dissection, neoadjuvant chemotherapy), and the patients were followed up at 3 and 5 years after surgery to compare the incidence of endpoint events (death and disease recurrence) and to identify associated risk factors.

### 2.2 Statistical Analyses

Univariate analysis: analysis of variance (ANOVA) is used. For cases involving theoretical frequencies < 5, Fisher's exact test is applied. For ordinal data, the Kruskal–Wallis H test is adopted. Kaplan–Meier method to assess differences in OS and RFS across various subsites. The log‐rank test determined statistical significance, with a *p* value < 0.05. Clinical pathological characteristics with *p* < 0.05 in univariate analysis (tobacco, T stage, N stage, differentiation grade, and neoadjuvant chemotherapy) were prioritized for inclusion in multivariate analysis. Additionally, factor (alcohol) potentially associated with the development of OSCC were also included into the analysis. Multivariate Cox proportional hazards model was used to identify clinicopathological factors influencing survival, calculating hazard ratios and 95% confidence intervals (95% CI). Analyses were conducted using SPSS R26.0.0.0 software.

## 3 Results

### 3.1 Demographics

This study retrospectively collected 1132 patients who were first admitted to our center and underwent surgical treatment from 2000 to 2020, and finally included 885 patients with complete medical record data and follow‐up data (median follow‐up time of 52.0 months [IQR: 17.0–96.0 months]) (Table 1). The patient population in this study was predominantly males over 50 years old, with 695 patients (78.5%) aged above 50 and 570 male patients (64.4%). Most patients had a history of tobacco exposure (*n* = 445, 50.3%), with the highest proportion observed in patients with floor of mouth cancer (*n* = 93, 65.5%). In contrast, the proportion of patients with a history of alcohol consumption was relatively low (*n* = 321, 36.3%). According to the seventh edition AJCC staging guidelines, 497 patients (56.2%) were diagnosed with Stages III–IV at initial diagnosis. Among these, the proportions of Stages III–IV in gingival cancer (*n* = 185, 65.8%) and floor of mouth cancer (*n* = 95, 66.9%) were significantly higher than those in other oral subsites. Consistent with their moderate to advanced stage distribution characteristics, these two subsites also had higher proportions of T3 + T4 stage tumors (gingival cancer: *n* = 148, 52.6%; floor of mouth cancer: *n* = 65, 45.7%) and lymph node metastasis rates (gingival cancer: *n* = 114, 40.6%; floor of mouth cancer: *n* = 74, 52.1%). Clinical observations showed that cervical lymph node metastases in patients with OSCC were mainly concentrated in Level I (*n* = 168, 47.3%) and Level II (*n* = 187, 52.7%). Among common oral cancer sites, floor of mouth cancer had the highest metastasis rate to Level I (*n* = 39, 52.7%), while gingival cancer exhibited relatively high metastasis rates to both Level I (*n* = 50, 43.9%) and Level II (*n* = 55, 48.2%). Notably, gingival cancer also had low‐probability metastases to Level IV (*n* = 6, 4.2%) and Level V (*n* = 5, 3.5%).

**TABLE 1:** Clinicopathological characteristics of patients with OSCC included in the study at our center from 2000 to 2020.

	Overal *n* = 885	Tumor subsite						*p*
		Oral tongue *n* = 371, 41.9%	Gingiva *n* = 281, 31.8%	Floor of mouth *n* = 142, 16.0%	Buccal mucosa *n* = 53, 6.0%	Retromolar trigone *n* = 20, 2.3%	Hard palate *n* = 18, 2.0%	
Age, *n* (%)								**0.001**
≤ 50	190 (21.5)	101 (27.2)	50 (17.8)	28 (19.7)	3 (5.6)	6 (30.0)	2 (11.1)	
> 50	695 (78.5)	270 (72.8)	231 (82.2)	114 (80.3)	50 (94.3)	14 (70.0)	16 (88.8)	
Gender, *n* (%)								**< 0.001**
Male	570 (64.4)	233 (62.8)	164 (58.4)	129 (90.8)	20 (37.7)	13 (65.0)	11 (61.1)	
Female	315 (35.6)	138 (37.2)	117 (41.6)	13 (9.2)	33 (62.3)	7 (35.0)	7 (38.9)	
Tobacco, *n* (%)								**< 0.001**
No	440 (49.7)	190 (51.2)	146 (52.0)	49 (34.5)	35 (66.0)	13 (65.0)	7 (38.9)	
Yes	445 (50.3)	181 (48.8)	135 (48.0)	93 (65.5)	18 (34.0)	7 (35.0)	11 (61.1)	
Alcohol, *n* (%)								**0.008**
No	564 (63.7)	234 (63.1)	192 (68.3)	73 (51.4)	40 (75.5)	14 (70.0)	11 (61.1)	
Yes	321 (36.3)	137 (36.9)	89 (31.7)	69 (48.6)	13 (24.5)	6 (30.0)	7 (38.9)	
T stage, *n* (%)								**< 0.001**
T0	4 (0.5)	2 (0.5)	—	—	2 (3.8)	—	—	
T1	304 (34.4)	139 (37.5)	93 (33.1)	45 (31.7)	14 (26.4)	5 (25.0)	8 (44.4)	
T2	235 (26.6)	138 (37.2)	40 (14.2)	32 (22.5)	16 (30.2)	5 (25.0)	4 (22.2)	
T3	134 (15.1)	56 (15.1)	33 (11.7)	32 (22.5)	11 (20.8)	2 (10.0)	0 (0.0)	
T4	208 (23.5)	36 (9.7)	115 (40.9)	33 (23.2)	10 (18.9)	8 (40.0)	6 (33.3)	
N stage, *n* (%)								0.060
N0	530 (59.9)	247 (66.6)	167 (59.4)	68 (47.9)	27 (50.9)	10 (50.0)	11 (61.1)	
N+	355 (40.1)	124 (33.4)	114 (40.6)	74 (52.1)	26 (49.1)	10 (50.0)	7 (38.9)	
AJCC stage, *n* (%)								**< 0.001**
Stage I–II	388 (43.8)	208 (56.1)	96 (34.1)	47 (33.1)	19 (35.8)	8 (40.0)	10 (55.6)	
Stage III–IV	497 (56.2)	163 (43.9)	185 (65.8)	95 (66.9)	34 (64.2)	12 (60.0)	8 (44.4)	
Lymph distribution, *n* (%)								
Level I	168 (47.3)	52 (41.9)	50 (43.9)	39 (52.7)	14 (53.8)	8 (80.0)	5 (71.4)	**0.001**
Level II	187 (52.7)	82 (66.1)	55 (48.2)	24 (32.4)	17 (65.4)	4 (40.0)	5 (71.4)	0.265
Level III	83 (23.4)	39 (31.5)	19 (16.7)	19 (25.7)	6 (23.1)	—	—	0.085
Level IV	18 (5.1)	10 (8.1)	6 (4.2)	2 (2.7)	—	—	—	0.700
Level V	8 (2.3)	3 (2.4)	5 (3.5)	—	—	—	—	0.471
Depth of invasion, *n* (%)								**< 0.001**
≤ 5 mm	415 (46.9)	190 (51.2)	122 (43.4)	62 (43.7)	22 (41.5)	8 (40.0)	11 (61.1)	
> 5 mm, ≤ 10 mm	216 (24.4)	108 (29.1)	52 (18.5)	33 (23.2)	14 (26.4)	7 (35.0)	2 (11.1)	
> 10 mm	254 (28.7)	73 (19.7)	107 (38.1)	47 (33.1)	17 (32.1)	5 (25.0)	5 (27.8)	
Differentiation, *n* (%)								0.469
Well	322 (36.4)	148 (39.9)	96 (34.2)	40 (28.2)	24 (45.3)	7 (35.0)	7 (38.9)	
Moderate	420 (47.5)	168 (45.3)	137 (48.8)	74 (52.1)	22 (41.5)	11 (55.0)	8 (44.4)	
Poor	143 (16.2)	55 (14.8)	48 (17.1)	28 (19.7)	7 (13.2)	2 (10.0)	3 (16.7)	
Lymph node dissection, *n* (%)								0.075
No	191 (21.6)	93 (25.1)	58 (20.6)	28 (19.7)	10 (18.9)	—	2 (11.1)	
Yes	694 (78.4)	278 (74.9)	223 (79.4)	114 (80.3)	43 (81.1)	20 (100.0)	16 (88.9)	
Neoadjuvant chemotherapy, *n* (%)								**< 0.001**
No	517 (58.4)	266 (71.7)	121 (43.1)	80 (56.3)	29 (54.7)	11 (55.0)	10 (55.6)	
Yes	368 (41.6)	105 (28.3)	160 (56.9)	62 (43.7)	24 (45.3)	9 (45.0)	8 (44.4)	

*Note*: Bold values emphasize the values with a *p*‐value < 0.05.

### 3.2 Kaplan–Meier Survival Curves

The 3‐year OS of OSCC was 68.8%, the 5‐year OS was 53.4%, the 3‐year RFS was 66.0%, and the 5‐year RFS was 63.4% (Table 2).

**TABLE 2:** The 3‐year and 5‐year OS and RFS of patients with OSCC in common subsites in our center.

	Overall	Tumor subsite			*p*
		Oral tongue	Gingiva	Floor of mouth	
Overall survival, OS					
3 years	68.8%	77.6%	63.0%	57.0%	**< 0.001**
5 years	53.4%	62.3%	49.8%	37.3%	**< 0.001**
Recurrence‐free survival, RFS					
3 years	66.0%	73.0%	59.1%	61.3%	**< 0.001**
5 years	63.4%	71.7%	55.5%	57.0%	**< 0.001**

*Note*: Bold values emphasize the values with a *p*‐value < 0.05.

Regarding the common subsites, the oral tongue had the best 3‐year and 5‐year OS (3‐year OS 77.6%, 5‐year OS 62.3%), followed by that from the gingiva (3‐year OS 63.0%, 5‐year OS 49.8%), and the worst was from the floor of mouth (3‐year OS 57.0%, 5‐year OS 37.3%). The Log‐rank test showed that there were statistically significant differences in OS among different subsites (3‐year *p* < 0.001, 5‐year *p* < 0.001). For RFS, the oral tongue also had the best outcome (3‐year RFS 73.0%, 5‐year RFS 71.7%), conspicuously, unlike OS, gingival cancer had the worst RFS (3‐year RFS 59.1%, 5‐year RFS 55.5%), and the RFS of floor of mouth cancer was in the middle (3‐year RFS 61.3%, 5‐year RFS 57.0%). The Log‐rank test also indicated that there was a statistically significant difference among different subsites (3‐year *p* < 0.001, 5‐year *p* < 0.001).

**FIGURE 1:**
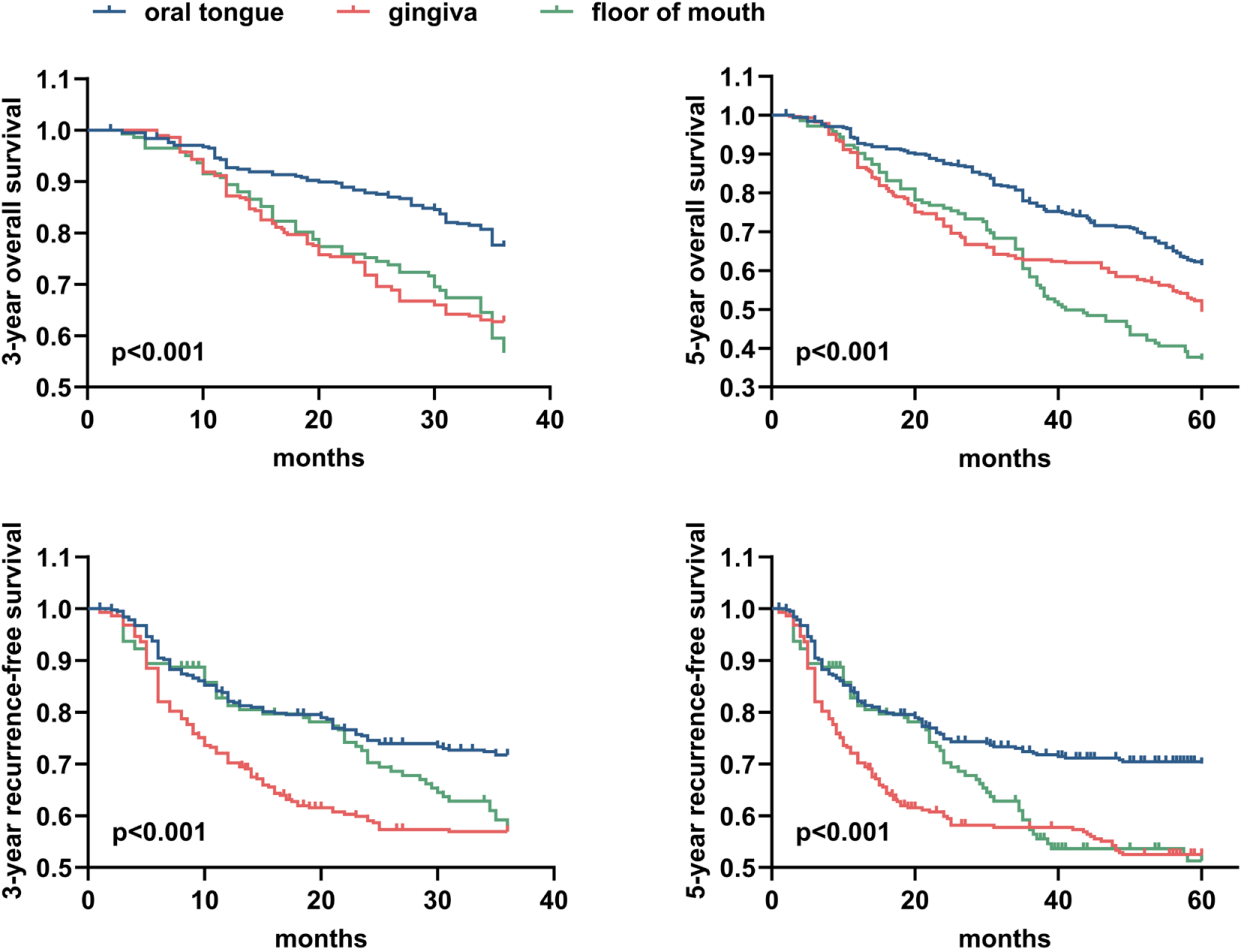
The Kaplan–Meier method was used to compare the prognoses among patients with OSCC in common subsites. The study revealed considerable variations in OS and RFS across different subsites of OSCC, such as the oral tongue, gingiva, and floor of the mouth. In terms of subsite‐specific prognosis, the oral tongue showed the most favorable outcomes, whereas the floor of mouth had the lowest OS, and the gingiva exhibited the poorest RFS. [Color figure can be viewed at wileyonlinelibrary.com]

Kaplan–Meier curve indicated that both the long‐term and short‐term OS of gingival cancer and floor of mouth cancer were significantly poorer than that of oral tongue cancer (Figure 1). Specifically, floor of mouth cancer began to exhibit poorer OS around 3 years postoperatively (3‐year OS: floor of mouth cancer 57.0%, gingival cancer 63.0%, oral tongue cancer 77.6%), and its 5‐year OS was significantly lower than those of the aforementioned two subsites (5‐year OS: floor of mouth cancer 37.3%, gingival cancer 49.8%, oral tongue cancer 62.3%). Notably, gingival cancer showed a high early postoperative recurrence rate, while floor of mouth cancer began to have a higher incidence of recurrence events around 2 years postoperatively.

### 3.3 Risk Factors Affecting the Prognosis of Gingival Cancer and Floor of Mouth Cancer

To further investigate the risk factors affecting the prognosis of these two subsites, the results of univariate analysis showed that T stage (gingival cancer: *p* < 0.001, floor of mouth cancer: *p* = 0.001), N stage (gingival cancer: *p* = 0.033, floor of mouth cancer: *p* = 0.001), depth of invasion (gingival cancer: *p* < 0.001, floor of mouth cancer: *p* = 0.028), and differentiation (gingival cancer: *p* < 0.001, floor of mouth cancer: *p* = 0.001) were significantly associated with patient survival. Notably, gender (*p* = 0.013), tobacco consumption (*p* = 0.005), and neoadjuvant chemotherapy (*p* = 0.032) were significantly associated with the survival of patients with floor of mouth cancer (Table 3). Subsequently, we performed a risk analysis using a multivariate Cox regression model on the independent factors that were significantly associated with patient survival in the univariate analysis. Considering that gender differences in the patient population might cause effect modification and confounding effects in the analysis of risk factors, gender stratification was conducted. The results showed that higher T stages (T3: hazard ratio [HR] = 2.826, 95% CI 1.542–5.180, *p* = 0.001; T4: HR = 3.002, 95% CI 1.830–4.925, *p* < 0.001), positive cervical lymph nodes (HR = 1.487, 95% CI 1.028–2.150, *p* = 0.035), and poorer differentiation grades (moderate differentiation: HR = 3.579, 95% CI 2.189–5.850, *p* < 0.001; poor differentiation: HR = 3.436, 95% CI 1.923–6.140, *p* < 0.001) were associated with the significantly increased risk of death in patients with gingival cancer. Notably, neoadjuvant chemotherapy (HR = 0.598, 95% CI 0.396–0.902, *p* = 0.014) were associated with the significantly reduced risk of death in patients with gingival cancer. In patients with floor of mouth cancer, tobacco consumption (HR = 1.952, 95% CI 1.141–3.337, *p* = 0.015) was an independent risk factor promoting patient death (Table 4).

**TABLE 3:** Univariate analysis of risk factors associated with death from gingival cancer and floor of mouth cancer.

	Gingiva				Floor of mouth			
	Total	Survival	Death	*p*	Total	Survival	Death	*p*
Age				0.222				0.811
≤ 50	50	21	29		28	11	17	
> 50	231	119	112		114	42	72	
Gender				0.369				**0.013**
Female	117	62	55		13	9	4	
Male	164	78	86		129	44	85	
Tobacco				0.135				**0.005**
No	146	79	67		49	26	23	
Yes	135	61	74		93	27	66	
Alcohol				0.171				0.794
No	192	101	91		73	28	45	
Yes	89	39	50		69	25	44	
T stage				**< 0.001**				**0.001**
T1	93	65	28		45	26	19	
T2	40	21	19		32	13	19	
T3	33	12	21		32	8	24	
T4	115	42	73		33	6	27	
N stage				**0.033**				**0.001**
N0	167	92	75		68	35	33	
N+	114	48	66		74	18	56	
Depth of invasion				**< 0.001**				**0.028**
≤ 5 mm	122	79	43		62	30	32	
> 5 mm, ≤ 10 mm	52	26	26		33	12	21	
> 10 mm	107	35	72		47	11	36	
Differentiation				**< 0.001**				**0.001**
Well	96	75	21		40	22	18	
Moderate	137	45	92		74	28	46	
Poor	48	20	28		28	3	25	
Lymph node dissection				0.393				0.499
No	58	26	32		28	12	16	
Yes	223	114	109		114	41	73	
Neoadjuvant chemotherapy				0.371				**0.032**
No	121	64	57		80	36	44	
Yes	160	76	84		62	17	45	

*Note*: Bold values emphasize the values with a *p*‐value < 0.05.

**TABLE 4:** Multivariate Cox regression analysis of independent risk factors associated with 5‐year OS from gingival cancer and floor of mouth cancer.

	Gingiva		Floor of mouth	
	HR (95% CI)	*p*	HR (95% CI)	*p*
Tobacco				
No	1		1	
Yes	0.737 (0.450–1.209)	0.227	1.952 (1.141–3.337)	**0.015**
Alcohol				
No	1		1	
Yes	1.528 (0.927–2.517)	0.096	0.974 (0.604–1.570)	0.914
T stage				
T1	1		1	
T2	1.442 (0.798–2.604)	0.225	1.174 (0.595–2.316)	0.644
T3	2.826 (1.542–5.180)	**0.001**	1.436 (0.731–2.819)	0.293
T4	3.002 (1.830–4.925)	**< 0.001**	2.543 (1.321–4.897)	**0.005**
N stage				
N0	1		1	
N+	1.487 (1.028–2.150)	**0.035**	1.576 (0.976–2.544)	0.063
Differentiation				
Well	1		1	
Moderate	3.579 (2.189–5.850)	**< 0.001**	1.412 (0.773–2.578)	0.262
Poor	3.436 (1.923–6.140)	**< 0.001**	1.987 (0.979–4.032)	0.057
Neoadjuvant chemotherapy				
No	1		1	
Yes	0.598 (0.396–0.902)	**0.014**	1.038 (0.644–1.672)	0.879

*Note*: Bold values emphasize the values with a *p*‐value < 0.05.

## 4 Discussion

The anatomical specificity of oral subsites and geographic variation in carcinogenic factors shape the diversity of the global disease landscape of OSCC. At our center, OSCC predominantly originates from the oral tongue (41.9%) and gingiva (31.8%), followed by the floor of mouth (16.0%) and buccal mucosa (6.0%). The retromolar trigone (2.3%) and hard palate (2.0%) are the least common sites of occurrence. Notably, the incidence of gingival cancer surpasses that of floor of mouth and buccal mucosa cancers, diverging significantly from subsite distributions in Europe, America, and South Asia [9, 10, 11, 12]. Research shows that in high‐HDI regions such as Europe and America, the floor of mouth is a prevalent site for OSCC. These regions also have elevated rates of tobacco and alcohol use, the primary risk factors for OSCC [11, 13, 14, 15]. In South Asia, OSCC predominantly affects the buccal mucosa, with high betel nut consumption cited as a key factor contributing to the region's highest global incidence of oral cancer [12, 16]. This is also consistent with the high incidence rates of buccal cancer in areca nut‐endemic areas in China [17, 18]. In this study, the reason for the high incidence of gingival cancer at our center was not observed, but it should not be easily ignored because another study in an area adjacent to our center also pointed out the high incidence of gingival cancer [19]. This may hint at potential region‐specific etiological factors within certain ecological and sociodemographic contexts.

This study also found that the proportion of patients with gingival cancer and floor of mouth cancer who were diagnosed at advanced stages during initial diagnosis was significantly higher than that of patients with oral tongue cancer; additionally, their prognostic performance was significantly poorer. This suggests that gingival cancer and floor of mouth cancer may have poorer biological behavior or be underscreened for tumors at these two sites, which has sparked our research interest in these subsites. Notably, gingival cancer has been reported to have a lower incidence in previous studies, leading to lower attention and limited understanding of tumors at this site. This makes it urgent for us to analyze the high‐risk factors affecting its prognosis. Univariate analysis revealed that T stage, N stage, DOI, and differentiation grade were all significantly associated with mortality in patients with gingival cancer, and this result aligns with the conclusions of previous studies [20]. Furthermore, in the multivariate analysis, we also observed that preoperative neoadjuvant chemotherapy for gingival cancer significantly reduced death risk. This underscores the importance of early screening, early diagnosis, and early systematic treatment for patients with gingival cancer. Although there is insufficient basis for determining that gingival cancer is highly malignant solely based on its disease stage at initial diagnosis, as its deeper anatomical location makes early screening difficult; compared with lip lesions, the probability of precancerous lesions in the gingiva progressing to malignant tumors is 6–10 times higher [18], and early symptoms are subtle, making it easy to be confused with benign diseases [21]. However, studies have clearly shown that there is no significant difference in the delayed diagnosis time between gingival cancer and cancers in other subsites of the oral cavity. Nevertheless, the frequency of invading adjacent bony tissue is significantly higher than that of cancers in other sites, which is associated with the thinness of the gingival mucosal tissue and its proximity to bony tissue [22]. Therefore, it is certain that enhancing attention to and screening for gingival cancers, as well as detecting cancer foci at an early stage, are crucial for improving the prognosis of patients with gingival cancer. This is particularly significant for patients with lesions that may progress to oral cancer, such as oral lichen planus, oral leukoplakia, and Fanconi's anemia [23].

Tobacco consumption has long been recognized as a high‐risk factor for oral cancer, but this association may be significantly correlated only with certain oral subsites [24]. A population‐based study in the United States over a 10‐year period showed that as smoking rates in the country declined, the incidence rates of only some oral cancer subsites decreased—including floor of mouth cancer and hard palate cancer [25]. The mechanisms by which tobacco induces oral cancer can be roughly categorized into two types: one involves physical damage (e.g., friction, thermal stimulation) from practices such as tobacco chewing, which triggers repeated inflammatory repair [26]; the other involves chemical‐induced base mutations caused by substances like reactive oxygen species (ROS) generated during tobacco use [27]. Additionally, studies have reported that oral dysbiosis (imbalance of oral microbiota) resulting from tobacco use is also one of the contributing factors to oral cancer [28]. Eloranta et al. [24] explicitly proposed that the carcinogenic effect of tobacco varies by oral subsite. Specifically, the floor of mouth, located at the lowest part of the oral cavity, is prone to the accumulation of carcinogens from tobacco. Moreover, it is covered by a thin layer of non‐keratinized epithelium, making it more sensitive to chemical and physical stimuli. Consequently, the association between carcinogenic effect of tobacco and floor of mouth cancer is the most pronounced. Consistent with these findings, our study also revealed that among patients with floor of mouth cancer, the proportion of those with a history of tobacco consumption was significantly higher than that in patients with OSCC at other subsites. Furthermore, we identified tobacco consumption as an independent risk factor affecting the prognosis of floor of mouth cancer. Therefore, for floor of mouth cancer, in addition to strengthening early screening, it is also essential to enhance smoking cessation education for high‐risk groups.

Beyond these anatomical and carcinogenic influences, genetic‐level differences may also underlie the high malignancy of gingival cancer and floor of mouth cancer. Eichberger et al. [29] observed that the expression of matrix metalloproteinase 27 (MMP‐27) was significantly lower in tumor tissues with mandibular bone invasion, compared with that in gingival and floor of mouth cancer tissues that grow along the mandible without invasion. This finding clarifies the key role of gene expression in tumor invasion; notably, it should be further compared with findings in oral tongue cancer tissues to highlight the unique role of MMP‐27 in driving the highly malignant progression of gingival and floor of mouth cancers. As a homologue of the tumor suppressor gene p53, p63 exerts a critical role in the pathogenesis of head and neck squamous cell carcinoma (HNSCC). A study investigating the baseline expression of p63 in normal tongue and gingival tissues revealed that the average p63 expression in normal tongue tissue was 2.5 times higher than that in normal gingival tissue. When normal tissues were compared with tumor tissues, p63 expression was significantly reduced in tongue cancer tissues, whereas no significant alterations were noted in gingival and floor of mouth cancer tissues. These changes in tumor suppressor genes may help account for the characteristic rapid progression of gingival and floor of mouth cancers [30].

Neoadjuvant chemotherapy has emerged as a pivotal downstaging strategy for locally advanced OSCC and demonstrated favorable therapeutic efficacy. In previous studies, 18%–43.2% of patients with unresectable advanced oral cancer have responded to the docetaxel–cisplatin two‐drug regimen or the three‐drug regimen combined with fluorouracil [31, 32, 33, 34], which provides an opportunity for surgical resection and improved long‐term prognosis in this subset of patients [35]. However, tumors at different subsites exhibit heterogeneous responses to this treatment [32]. Such variability is attributed to inherent differences in tissue architecture, epigenetic profiles, and other biological characteristics. Studies have shown that oral tumors deficient in functional p53 rarely attain a complete response to cisplatin/fluorouracil‐based chemotherapy, whereas 40% of patients whose tumors express functional p53 achieve complete response [36, 37]. Notably, the functional status of p53 has been proven to differ significantly across distinct oral cancer subsites [38]. Our study indicates that neoadjuvant chemotherapy is associated with improved prognosis in gingival cancer, but no such correlation has been found in floor of mouth cancer. This suggests the specificity of gingival cancer in the response to neoadjuvant chemotherapy. However, there is still limited research on the causes of this specificity. In the future, more cases should be included to comprehensively compare the neoadjuvant treatment response characteristics among different subsites. Additionally, multi‐omics data analysis should be performed to explore whether there are subsite‐specific factors that affect the bioavailable concentration of drugs in target tissues or drug resistance regulatory pathways.

The innovation of this study lies in being the first to compare the clinical characteristics of OSCC across different subsites in the region where our center is located. Additionally, it conducts long‐term follow‐up in a large cohort to evaluate the prognosis of different subsites, and performs risk factor analysis for mortality in subsites with high incidence and mortality rates, providing guidance for the refined management of oral cancer patients in this region. Meanwhile, this study also has certain limitations and requires further improvement. First, it is a single‐center study and lacks validation with multi‐center data. In subsequent research, multi‐center data should be included, particularly from centers in northern China with similar living environments and habits, to further explore the pathogenic characteristics of each subsite, especially the reasons for the high incidence of gingival cancer. Second, this study used the seventh edition TNM staging system and lacked data on prognostic factors such as neurovascular invasion and extranodal extension of lymph nodes when analyzing disease characteristics. While it identified and discussed differences in disease characteristics among subsites when comparing their survival characteristics, it lacked complete information on these confounding factors, primarily due to limitations in the details provided by pathologists in their descriptions of intraoperative pathological findings. In the future, detailed analysis of intraoperative pathological findings should be conducted, and prognostic differences across subsites should be re‐evaluated after strictly controlling for confounding factors. As a retrospective study, we were unable to obtain detailed data on patients' social determinants of health, such as socioeconomic status, distance to medical facilities, and health literacy. These factors may contribute to the differences in diagnostic staging among oral subsites. Future studies should incorporate these key variables to more accurately clarify the independent relationship between primary tumor subsites and their staging.

In conclusion, there are differences in disease characteristics among OSCC at different subsites. Furthermore, gingival cancer and floor of mouth cancer account for a relatively high proportion among OSCC; compared with oral tongue cancer, both exhibits more severe tumor invasion and lymph node metastasis at initial diagnosis. In terms of prognosis, both have significantly poorer outcomes than oral tongue cancer: gingival cancer shows a higher early postoperative recurrence rate, while floor of mouth cancer presents poorer OS, particularly during long‐term follow‐up. Regarding the underlying reasons, we identified that neoadjuvant chemotherapy is an independent protective factor for reducing mortality in patients with gingival cancer. Therefore, in the clinical management of patients with gingival cancer, priority should be given to enhancing patient adherence to neoadjuvant therapy and ensuring the delivery of complete, standardized treatment regimens. Additionally, during the early postoperative period of gingival cancer, especially within 2 years after surgery, close imaging monitoring should be conducted to promptly intervene in cases of recurrent gingival cancer. For patients with floor of mouth cancer, smoking cessation education is of great importance. Early reduction in tobacco consumption may significantly improve their prognosis.

## Author Contributions

Y.S.D., C.J., and X.D.W. conceived and designed the study. H.L., Y.S.W., A.L.Y., J.Z., and R.Y.S. did case collection and follow‐up. H.L., Y.S.D., Y.F., X.H.L., W.F.S., and S.S.C. contributed to data analysis. The manuscript was written by H.L. and Y.D. and revised by Y.S.W. and Y.S.D. Y.S.D, X.D.W. supervised the research. All authors read and approved the final manuscript.

## Funding

Peak Discipline Support Program of the 14th Five‐Year Plan of Tianjin Medical University Cancer Institute & Hospital (7‐2‐8).

## Ethics Statement

This study was approved by the Ethics Committee of Tianjin Medical University Cancer Institute & Hospital (approval number: bc20255237).

## Conflicts of Interest

The authors declare no conflicts of interest.

## Data Availability

The data that support the findings of this study are available from the corresponding author upon reasonable request.

## References

[1] W. Cao , K. Qin , F. Li , and W. Chen , “Socioeconomic Inequalities in Cancer Incidence and Mortality: An Analysis of GLOBOCAN 2022,” Chinese Medical Journal 137 (2024): 1407–1413. 38616547 10.1097/CM9.0000000000003140 PMC11188912

[2] P. Tandon , A. Dadhich , H. Saluja , S. Bawane , and S. Sachdeva , “The Prevalence of Squamous Cell Carcinoma in Different Sites of Oral Cavity at Our Rural Health Care Centre in Loni, Maharashtra—A Retrospective 10‐Year Study,” Contemporary Oncology (Poznan, Poland) 21 (2017): 178–183. 28947890 10.5114/wo.2017.68628 PMC5611509

[3] S. He , C. Xia , H. Li , et al., “Cancer Profiles in China and Comparisons With the USA: A Comprehensive Analysis in the Incidence, Mortality, Survival, Staging, and Attribution to Risk Factors,” Science China. Life Sciences 67 (2024): 122–131. 37755589 10.1007/s11427-023-2423-1

[4] L. Xie , C. M. Huang , Y. L. Song , Z. Shao , and Z. J. Shang , “Incidence Trends and Projections of Lip and Oral Cavity Cancer in China 1990‐2021: An Age‐Period‐Cohort and Decomposition Analysis,” BMC Oral Health 25 (2025): 406. 40108625 10.1186/s12903-025-05764-2 PMC11924705

[5] C. Kirsch , “Oral Cavity Cancer,” Topics in Magnetic Resonance Imaging 18 (2007): 269–280. 17893592 10.1097/RMR.0b013e3181572caa

[6] S. L. Zhang , J. W. Tian , J. Jia , and Z. L. Yu , “A New Approach: Cervical Approach for Marginal Resection of the Posterior Mandible,” Journal of Stomatology Oral and Maxillofacial Surgery 126 (2025): 102046. 39251069 10.1016/j.jormas.2024.102046

[7] S. J. La'porte , J. K. Juttla , and R. K. Lingam , “Imaging the Floor of the Mouth and the Sublingual Space,” Radiographics 31 (2011): 1215–1230. 21918039 10.1148/rg.315105062

[8] A. Grammatica , C. Piazza , M. Ferrari , et al., “Step‐By‐Step Cadaver Dissection and Surgical Technique for Compartmental Tongue and Floor of Mouth Resection,” Frontiers in Oncology 11 (2021): 613945. 33968719 10.3389/fonc.2021.613945 PMC8104033

[9] A. Miranda‐Filho and F. Bray , “Global Patterns and Trends in Cancers of the Lip, Tongue and Mouth,” Oral Oncology 102 (2020): 104551. 31986342 10.1016/j.oraloncology.2019.104551

[10] Z. Farhood , M. Simpson , G. M. Ward , R. J. Walker , and N. Osazuwa‐Peters , “Does Anatomic Subsite Influence Oral Cavity Cancer Mortality? A SEER Database Analysis,” Laryngoscope 129 (2019): 1400–1406. 30408182 10.1002/lary.27490

[11] M. M. Justesen , H. Stampe , K. K. Jakobsen , et al., “Impact of Tumor Subsite on Survival Outcomes in Oral Squamous Cell Carcinoma: A Retrospective Cohort Study From 2000 to 2019,” Oral Oncology 149 (2024): 106684. 38211527 10.1016/j.oraloncology.2024.106684

[12] S. Warnakulasuriya and A. M. Filho , “Oral Cancer in the South and South‐East Asia Region, 2022: Incidence and Mortality,” Oral Diseases 31 (2025): 1398–1405. 40364456 10.1111/odi.15369

[13] B. V. Sundermann , L. Uhlmann , J. Hoffmann , K. Freier , and O. C. Thiele , “The Localization and Risk Factors of Squamous Cell Carcinoma in the Oral Cavity: A Retrospective Study of 1501 Cases,” Journal of Cranio‐Maxillo‐Facial Surgery 46 (2018): 177–182. 29242026 10.1016/j.jcms.2017.10.019

[14] Y. Li , J. Tian , Y. You , et al., “Global Variations and Socioeconomic Inequalities in Lifetime Risk of Lip, Oral Cavity, and Pharyngeal Cancer: A Population‐Based Systematic Analysis of GLOBOCAN 2022,” International Journal of Surgery 111 (2025): 3698–3709. 40277389 10.1097/JS9.0000000000002408 PMC12165512

[15] B. A. van Dijk , M. T. Brands , S. M. Geurts , M. A. Merkx , and J. L. Roodenburg , “Trends in Oral Cavity Cancer Incidence, Mortality, Survival and Treatment in The Netherlands,” International Journal of Cancer 139 (2016): 574–583. 27038013 10.1002/ijc.30107

[16] J. Qiu , H. Wen , J. Bai , and C. Yu , “The Mortality of Oral Cancer Attributable to Tobacco in China, the US, and India,” Journal of Cancer Research and Clinical Oncology 149 (2023): 16741–16752. 37728701 10.1007/s00432-023-05400-y PMC11797500

[17] Y. Hu , R. Zhong , H. Li , and Y. Zou , “Effects of Betel Quid, Smoking and Alcohol on Oral Cancer Risk: A Case‐Control Study in Hunan Province, China,” Substance Use & Misuse 55 (2020): 1501–1508. 32569534 10.1080/10826084.2020.1750031

[18] W. W. Su , C. W. Su , D. C. Chang , et al., “Impact of Varying Anatomic Sites on Advanced Stage and Survival of Oral Cancer: 9‐Year Prospective Cohort of 27 717 Cases,” Head & Neck 41 (2019): 1475–1483. 30652378 10.1002/hed.25579

[19] X. X. Bai , J. Zhang , and L. Wei , “Analysis of Primary Oral and Oropharyngeal Squamous Cell Carcinoma in Inhabitants of Beijing, China—A 10‐Year Continuous Single‐Center Study,” BMC Oral Health 20 (2020): 208. 32680501 10.1186/s12903-020-01192-6 PMC7367409

[20] S. Yoshida , T. Shimo , Y. Murase , et al., “The Prognostic Implications of Bone Invasion in Gingival Squamous Cell Carcinoma,” Anticancer Research 38 (2018): 955–962. 29374727 10.21873/anticanres.12309

[21] E. Chimenos Küstner , F. Finestres Zubeldia , and P. Huguet Redecilla , “Gingival Squamous Cell Carcinoma: A Clinical Case and Differential Diagnosis,” Medicina Oral 6 (2001): 335–341. 11694866

[22] J. Seoane , P. I. Varela‐Centelles , T. F. Walsh , J. L. Lopez‐Cedrun , and I. Vazquez , “Gingival Squamous Cell Carcinoma: Diagnostic Delay or Rapid Invasion?,” Journal of Periodontology 77 (2006): 1229–1233. 16805687 10.1902/jop.2006.050408

[23] Y. Smail , M. Troizier‐Cheyne , C. M. Lutz , and A. L. Ejeil , “Clinico‐Pathological Specificities of Gingival Carcinoma Among 32 Patients With Oral Cancer: A Cross Sectional Retrospective and Observational Study,” BMC Oral Health 24 (2024): 1317. 39472880 10.1186/s12903-024-05078-9 PMC11523822

[24] R. Eloranta , S. T. Vilén , A. Keinänen , et al., “Oral Squamous Cell Carcinoma: Effect of Tobacco and Alcohol on Cancer Location,” Tobacco Induced Diseases 22 (2024): 1–9. 10.18332/tid/189303 PMC11185050 38895166

[25] T. D. Ellington , S. J. Henley , V. Senkomago , et al., “Trends in Incidence of Cancers of the Oral Cavity and Pharynx—United States 2007‐2016,” MMWR. Morbidity and Mortality Weekly Report 69 (2020): 433–438. 32298244 10.15585/mmwr.mm6915a1 PMC7755056

[26] P. B. Sehgal , H. Yuan , and S. V. DiSenso‐Browne , “Temperature and WNK‐SPAK/OSR1 Kinases Dynamically Regulate Antiviral Human GFP‐MxA Biomolecular Condensates in Oral Cancer Cells,” Cells 14 (2025): 947. 40643468 10.3390/cells14130947 PMC12248889

[27] India Project Team of the International Cancer Genome Consortium , “Mutational Landscape of Gingivo‐Buccal Oral Squamous Cell Carcinoma Reveals New Recurrently‐Mutated Genes and Molecular Subgroups,” Nature Communications 4 (2013): 2873. 10.1038/ncomms3873 PMC3863896 24292195

[28] A. Sami , I. Elimairi , C. A. Ryan , C. Stanton , D. Patangia , and R. P. Ross , “Altered Oral Microbiome in Sudanese Toombak Smokeless Tobacco Users Carries a Newly Emerging Risk of Squamous Cell Carcinoma Development and Progression,” Scientific Reports 13 (2023): 6645. 37095112 10.1038/s41598-023-32892-y PMC10125980

[29] J. Eichberger , F. Weber , G. Spanier , et al., “Loss of MMP‐27 Predicts Mandibular Bone Invasion in Oral Squamous Cell Carcinoma,” Cancers (Basel) 14 (2022): 4044. 36011038 10.3390/cancers14164044 PMC9406335

[30] L. Boldrup , P. J. Coates , G. Laurell , and K. Nylander , “Differences in p63 Expression in SCCHN Tumours of Different Sub‐Sites Within the Oral Cavity,” Oral Oncology 47 (2011): 861–865. 21802344 10.1016/j.oraloncology.2011.07.002

[31] V. M. Patil , K. Prabhash , V. Noronha , et al., “Neoadjuvant Chemotherapy Followed by Surgery in Very Locally Advanced Technically Unresectable Oral Cavity Cancers,” Oral Oncology 50 (2014): 1000–1004. 25130412 10.1016/j.oraloncology.2014.07.015

[32] J. Y. Fu , X. H. Yue , M. J. Dong , J. Li , and C. P. Zhang , “Assessment of Neoadjuvant Chemotherapy With Docetaxel, Cisplatin, and Fluorouracil in Patients With Oral Cavity Cancer,” Cancer Medicine 12 (2023): 2417–2426. 35880556 10.1002/cam4.5075 PMC9939210

[33] V. Noronha , A. Dhanawat , V. M. Patil , et al., “Long‐Term Outcomes of Neo‐Adjuvant Chemotherapy on Borderline Resectable Oral Cavity Cancers: Real‐World Data of 3266 Patients and Implications for Clinical Practice,” Oral Oncology 148 (2024): 106633. 37988838 10.1016/j.oraloncology.2023.106633

[34] A. Joshi , V. M. Patil , V. Noronha , et al., “Is There a Role of Induction Chemotherapy Followed by Resection in T4b Oral Cavity Cancers?,” Indian Journal of Cancer 50 (2013): 349–355. 24369216 10.4103/0019-509X.123627

[35] P. Bossi , S. Lo Vullo , M. Guzzo , et al., “Preoperative Chemotherapy in Advanced Resectable OCSCC: Long‐Term Results of a Randomized Phase III Trial,” Annals of Oncology 25 (2014): 462–466. 24401930 10.1093/annonc/mdt555

[36] F. Perrone , P. Bossi , B. Cortelazzi , et al., “TP53 Mutations and Pathologic Complete Response to Neoadjuvant Cisplatin and Fluorouracil Chemotherapy in Resected Oral Cavity Squamous Cell Carcinoma,” Journal of Clinical Oncology 28 (2010): 761–766. 20048189 10.1200/JCO.2009.22.4170

[37] E. A. Mroz and J. W. Rocco , “Functional p53 Status as a Biomarker for Chemotherapy Response in Oral‐Cavity Cancer,” Journal of Clinical Oncology 28 (2010): 715–717. 20048171 10.1200/JCO.2009.26.3475

[38] B. Ravishankar , B. V. Madhavi , A. Kalagara , et al., “Clinical and Pathological Correlation of P(53) Expression in Oral Cancers,” Pathology, Research and Practice 253 (2024): 155071. 38181580 10.1016/j.prp.2023.155071

